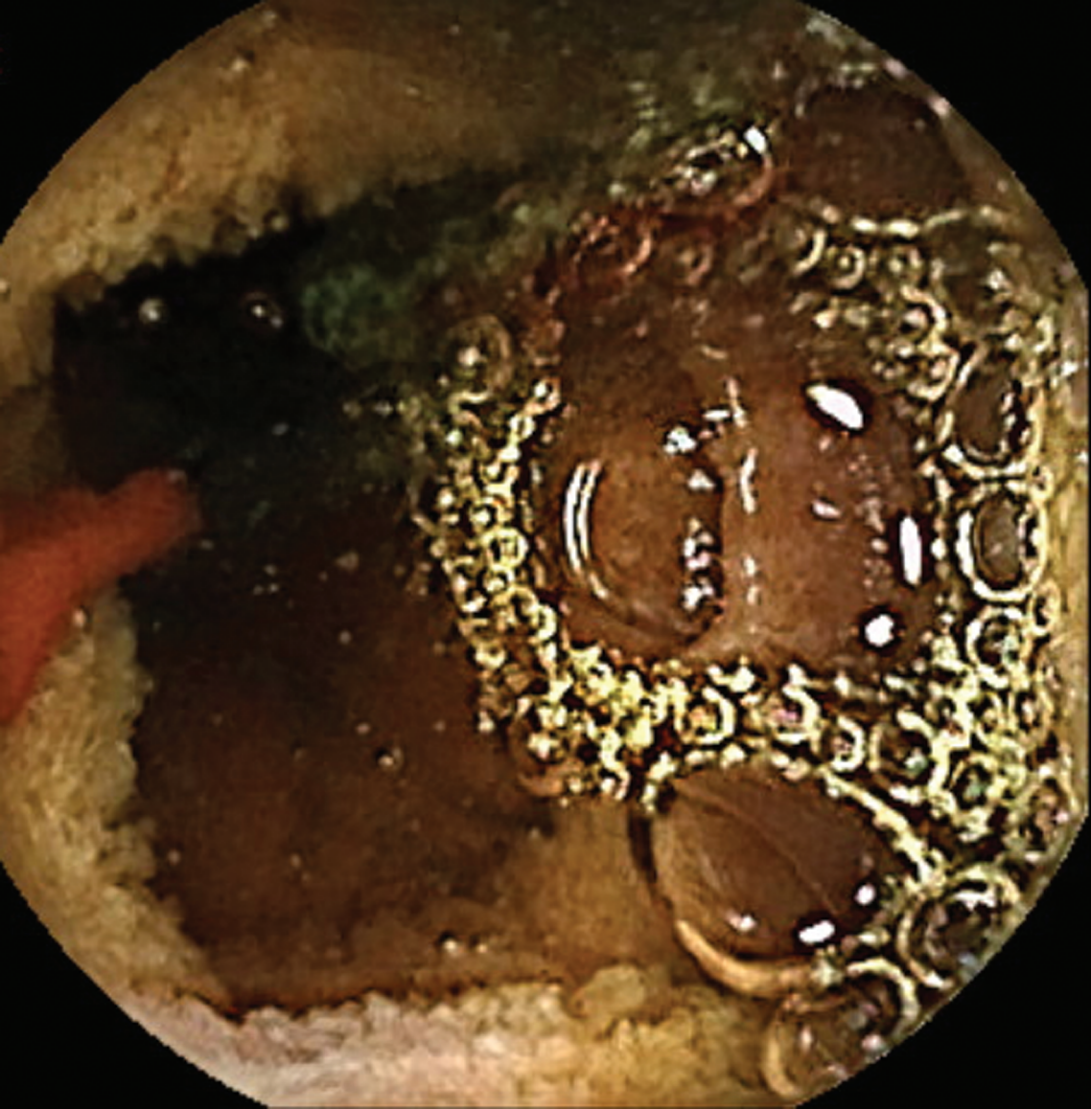# An Actively Bleeding Lesion in Video Capsule Endoscopy Study as a Source of Occult Gastrointestinal Bleeding

**DOI:** 10.1093/jcag/gwz008

**Published:** 2019-05-10

**Authors:** Mohammad Yaghoobi, Bruno Salena

**Affiliations:** 1 Division of Gastroenterology, Michael G. DeGroote School of Medicine, McMaster University and McMaster University Medical Center, Hamilton, Ontario, Canada; 2 Farncombe Family Digestive Health Research Institute, McMaster University, Hamilton, Ontario, Canada

**Fig 1:**
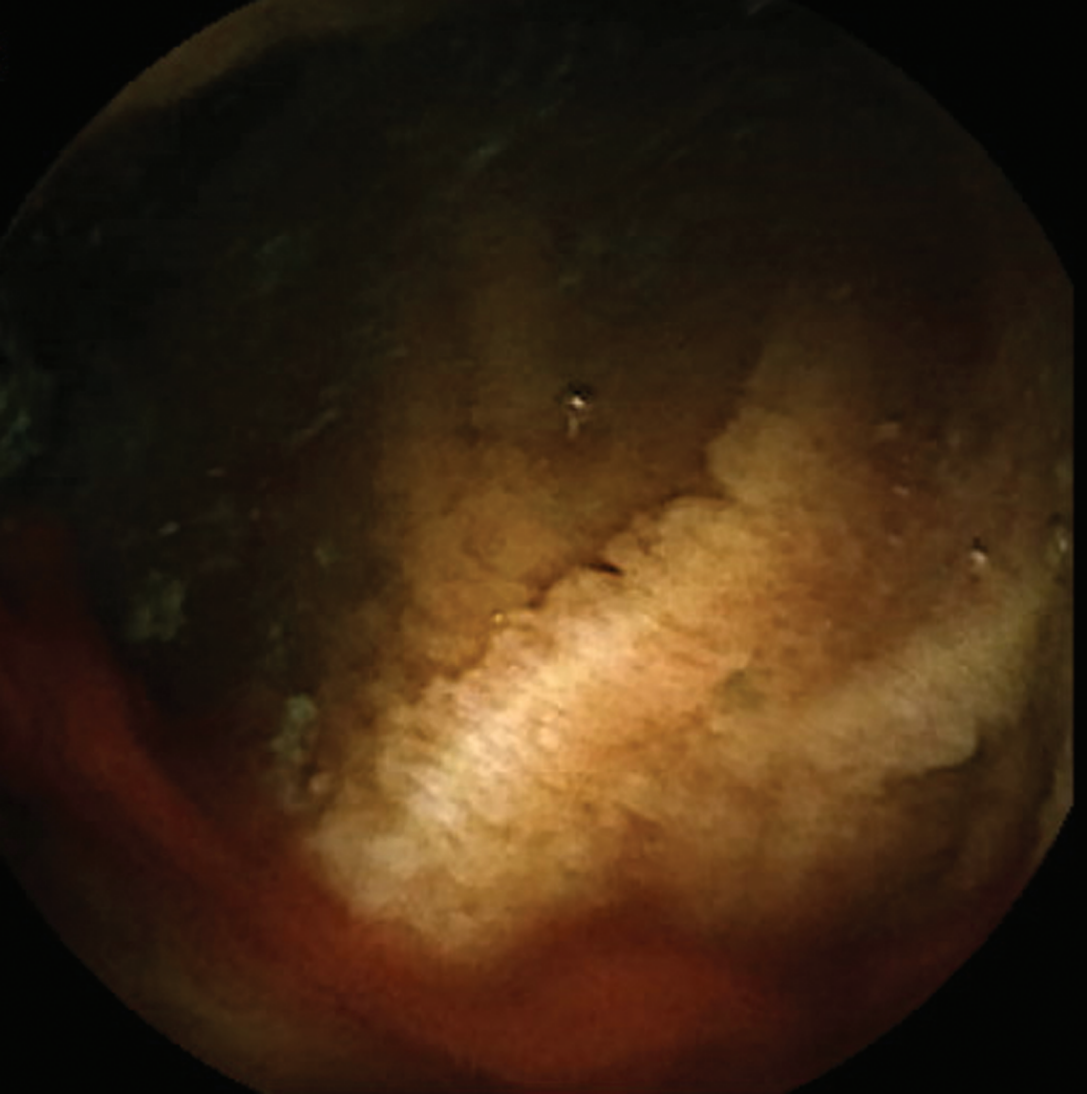


**Fig 2:**
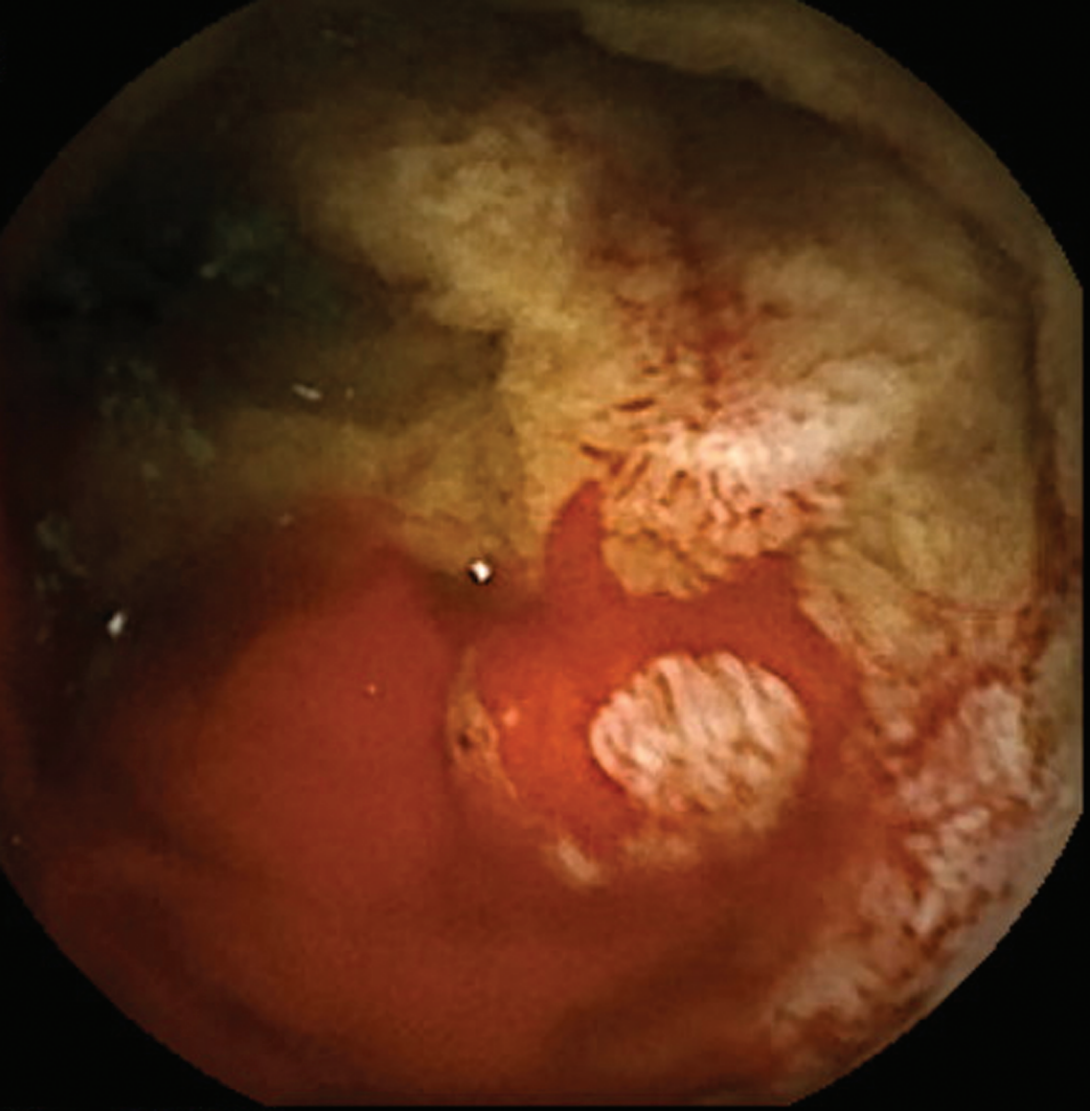


**Fig 3:**
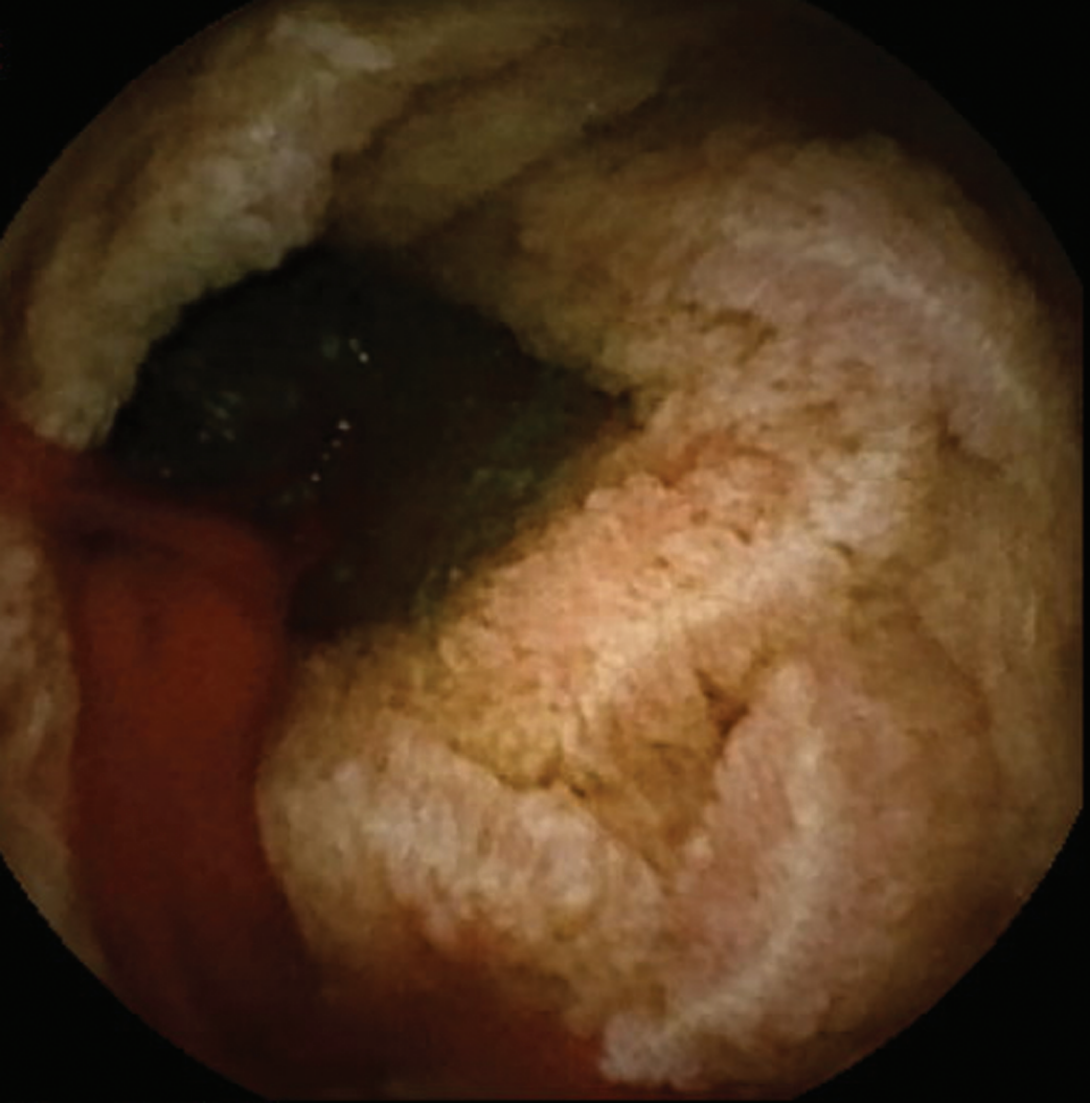


An 81-year-old gentleman with longstanding history of transfusion-dependent anemia and occult gastrointestinal bleeding since 2014 was referred for a video capsule endoscopy (VCE) study. His past medial history included chronic myeloid leukemia, splenectomy, spinal stenosis, hypertension, diabetes mellitus type II, dyslipidemia and a cerebrovascular accident. Family history was positive for colorectal carcinoma in brother at the age of 60. The anemia was first diagnosed after a coronary artery bypass graft for coronary artery disease in 2014. He underwent full gastrointestinal workup including a VCE study in 2014 which was reported as normal. The patient was seen by a hematologist and was also diagnosed with myelodysplastic syndrome and was treated with oral and intravenous iron. He was then referred back to gastroenterology with melena. The initial VCE study revealed two actively bleeding lesion in distal jejunum and proximal ileum (Figures 1-4). The patient was referred for a double balloon-enteroscopy for the treatment of the bleeding lesions, however, the enteroscope could not be passed through the splenic flexure likely due to adhesions. A repeat VCE study revealed persistent bleeding from the lesion and a nonbleeding angiodysplasia. The patient continued to have bleeding requiring ongoing transfusion and admissions. He then underwent a provocation angiogram of the celiac axis and superior mesenteric artery which did not show any active bleeding. A Tc 99-m Meckel’s diverticulum study was also negative. He was eventually referred for an intraoperative enteroscopy and is waiting the procedure.

**Fig 4:**